# Red Blood Cell Destruction in Autoimmune Hemolytic Anemia: Role of Complement and Potential New Targets for Therapy

**DOI:** 10.1155/2015/363278

**Published:** 2015-01-29

**Authors:** Sigbjørn Berentsen, Tatjana Sundic

**Affiliations:** ^1^Department of Medicine, Haugesund Hospital, Helse Fonna, P.O. Box 2170, 5504 Haugesund, Norway; ^2^Department of Immunology and Transfusion Medicine, Haugesund Hospital, Helse Fonna, P.O. Box 2170, 5504 Haugesund, Norway

## Abstract

Autoimmune hemolytic anemia (AIHA) is a collective term for several diseases characterized by autoantibody-initiated destruction of red blood cells (RBCs). Exact subclassification is essential. We provide a review of the respective types of AIHA with emphasis on mechanisms of RBC destruction, focusing in particular on complement involvement. Complement activation plays a definitive but limited role in warm-antibody AIHA (w-AIHA), whereas primary cold agglutinin disease (CAD), secondary cold agglutinin syndrome (CAS), and paroxysmal cold hemoglobinuria (PCH) are entirely complement-dependent disorders. The details of complement involvement differ among these subtypes. The theoretical background for therapeutic complement inhibition in selected patients is very strong in CAD, CAS, and PCH but more limited in w-AIHA. The optimal target complement component for inhibition is assumed to be important and highly dependent on the type of AIHA. Complement modulation is currently not an evidence-based therapy modality in any AIHA, but a number of experimental and preclinical studies are in progress and a few clinical observations have been reported. Clinical studies of new complement inhibitors are probably not far ahead.

## 1. Introduction

Autoimmune hemolytic anemia (AIHA) is a heterogeneous group of disorders characterized by autoantibody-mediated destruction of red blood cells (RBCs) [1–3]. AIHA can be classified as shown in Table 1. Correct subclassification and identification of any underlying or associated disorder are critical for understanding the pathogenesis and for optimal therapeutic management [3–5].

The knowledge of etiology and pathogenesis, including details of RBC breakdown, is rapidly growing [3–7]. During the last five decades we have learned a great deal about the essential role of complement in subgroups of AIHA [6–8]. This insight is still expanding and possible therapeutic options for complement modulation are being explored [9–11]. Furthermore, though paroxysmal nocturnal hemoglobinuria (PNH) is not an autoimmune disorder, the entirely complement-dependent pathogenesis and the success of therapeutic complement inhibition in this disease make it possible to learn lessons from PNH that might prove useful in treating AIHA [12].

In this review, we will address the pathogenetic mechanisms of AIHA, focusing in particular on the role of complement for RBC destruction and possible implications for the potential therapeutic use of complement modulators. Established therapies will be briefly mentioned since they have relevance for future therapeutic perspectives. Diagnostic procedures will not be described as such; comprehensive guidelines for diagnosis can be found elsewhere in the literature [4, 5].

## 2. Warm-Antibody Autoimmune Hemolytic Anemia

### 2.1. Etiology, Pathogenesis, and Associated Disorders

The incidence of AIHA has been estimated to be about 1 : 100 000 per year in adults [13] and even lower in children. Warm-antibody AIHA (w-AIHA) accounts for approximately 75% of the cases [1, 2]. The autoantibodies in w-AIHA have temperature optimum at 37°C and are invariably polyclonal, even when w-AIHA complicates a clonal B-cell lymphoproliferative disorder [14, 15]. A general dysregulation of the immune system with impaired distinction between self and nonself seems essential to pathogenesis; the T-cell mediated regulation of the humoral immune system has been shown to play a critical role [15, 16]. Polymorphism of the gene for the signal substance CTLA-4, which activates regulatory T-cells (T_reg_-cells), seems to bring about a disposition for autoimmunity [16]. CD4^+^CD25^+^T_reg_-cells are important for immunological tolerance and, thereby, for preventing w-AIHA and other polyclonal autoimmune disorders [16].

On this background it is not surprising that a large number of immunological and lymphoproliferative disorders can be associated with w-AIHA. Secondary AIHA, that is, cases with a demonstrable associated or underlying disease, accounts for about 50% of w-AIHA, while the remaining 50% are classified as primary. The most frequently occurring associated lymphoproliferative disease is chronic lymphatic leukemia (CLL), whereas w-AIHA complicating another non-Hodgkin's lymphoma (NHL) is less common [1, 2, 14]. Examples of immunological disorders that can be associated with w-AIHA are systemic lupus erythematosus, rheumatoid arthritis, Sjögren's syndrome, primary biliary cirrhosis, hypothyroidism, inflammatory bowel disease, immune thrombocytopenia, and primary hypogammaglobulinemia [1, 2, 15, 17]. Some patients have several associated diseases at the same time.

Autoantibody or complement fragment deposition on the RBC can be detected using polyspecific and monospecific direct antiglobulin test (DAT). The findings by monospecific DAT reflect, although not to a completely reliable extent, which immunoglobulin class(es) or complement fragments are present on the RBC surface. The autoantibodies in w-AIHA are of the immunoglobulin G (IgG) class in most cases [4]. In up to 50% of w-AIHA, DAT is positive for complement fragments, most often C3d and usually in combination with IgG. IgA autoantibodies occur in 15–20% of the patients, either in combination with IgG or, more rarely, alone [18]. Cases with IgA as the sole autoantibody class may be misdiagnosed because reagents used in the polyspecific DAT do not usually contain anti-IgA. Warm autoantibodies of the IgM class have been assumed to be rare. Their frequency remains somewhat controversial, however, because they may have low affinity to the antigen and may have detached from the RBC surface before they can be detected by DAT [19, 20].

In 3–10% of patients with w-AIHA, DAT is found to be negative [4, 21]. The most established explanation is IgG deposition on RBC below the sensitivity threshold for DAT or, less frequently, occurrence of IgA as the only autoantibody class [4, 18]. The hypothesis that T- or NK-cell mediated immunity can destroy erythrocytes without involving the humoral immune system has been supported by a few casuistic and experimental observations [6, 22]. Furthermore, some evidence has been provided that Fc*γ*RI receptors on macrophages bind monomeric serum-IgG which can include low-concentration specific RBC antibodies. This will create “armed macrophages” carrying RBC antibodies whose Fab portion can react with nonsensitized RBC [23]. “DAT-negative AIHA” represents a diagnostic challenge. Elution techniques and flow cytometric methods may be of some value but in clinical practice, often, DAT-negative AIHA remains an exclusion diagnosis. The usefulness of newer gel centrifugation tests for IgG, IgG subclasses, and complement fragments in detecting autoimmune pathogenesis of hemolytic anemia was explored in a recent study. DAT strength remained the best diagnostic indicator for AIHA and had the strongest association with AIHA compared with other commercially available immunohematology tests [24].

### 2.2. Mechanisms of Erythrocyte Destruction

RBCs coated with warm-reactive autoantibodies are sequestered and phagocytosed by macrophages, primarily in the spleen [25–27]. The macrophage surface expresses receptors for the Fc region of the immunoglobulin molecules, which enables trapping and ingestion of the opsonized RBCs [28, 29]. Often, however, phagocytosis is incomplete and results in formation of spherocytes [7, 28]. This has been explained in part by the removal of more membranes than volume. In addition, ectoenzymes on the macrophage surface cause microperforations of the RBC membrane, increasing its permeability and thereby promoting the transition from a biconcave to a spherical shape of the cell [7, 25, 28]. Spherocytes are prone to further destruction during subsequent passages through the spleen. The severity of hemolysis correlates with the degree of spherocytosis, but not with the strength of DAT positivity [4, 7, 26].

**Figure 1:**
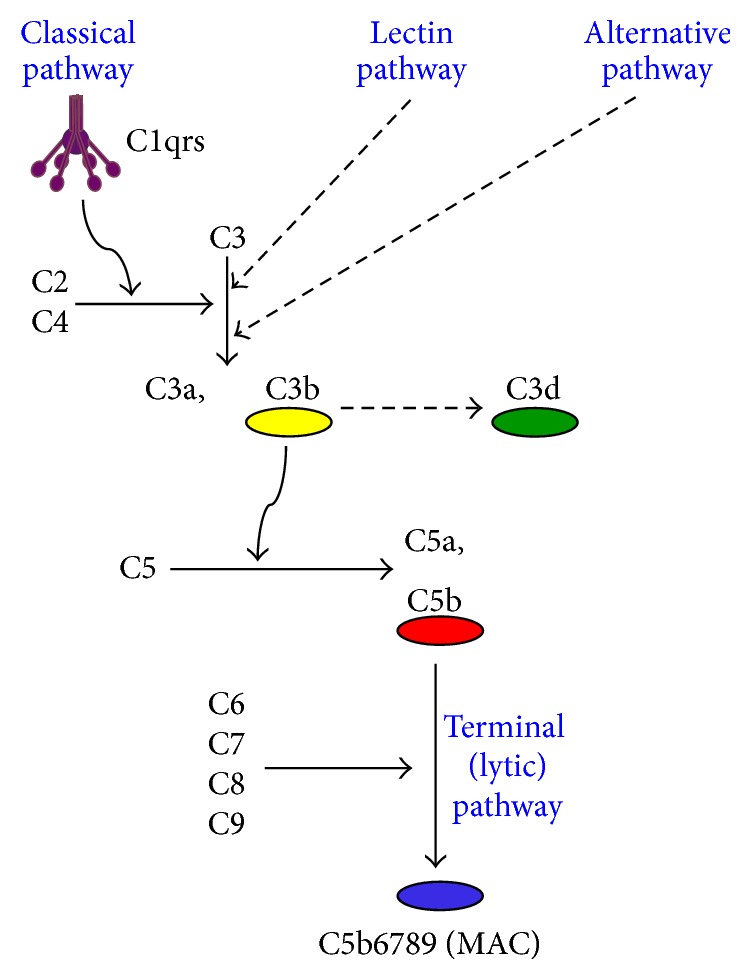
The complement cascade, simplified. Only components relevant for this paper are shown. The lectin and alternative pathways, converging with the classical pathway at the C3 activation level, are indicated but not shown in detail. Physiological inhibitors and positive feedback loops are not shown. C: complement protein; MAC: membrane attack complex.

On RBCs heavily coated with immunoglobulin, the amount of antigen-antibody complex can be sufficient for binding complement protein complex C1 and, thereby, for activation of the classical complement pathway (Figure 1) [30–32]. Unlike IgG, IgM is a potent complement activator but, as previously mentioned, is usually not found on the RBC surface by DAT in w-AIHA [19]. Regarding the IgG subclasses, IgG3 activates complement more efficiently than does IgG1, while IgG2 is a weak activator and there is no good evidence for complement activation by IgG4 [33, 34]. IgA does not probably activate complement. Despite this, however, IgA-deposition on RBC can lead to fulminant hemolysis [18, 35]. A probable explanation is involvement of IgM even in some cases where only IgG or IgA is detected, since IgM will often detach from the RBC before it can be detected by DAT [20]. At least in IgA-induced hemolysis, hemagglutination* per se* has also been shown to play a role. Upon complement activation in w-AIHA, phagocytosis of C3b-opsonized erythrocytes by Kupfer cells in the liver is responsible for most of the RBC destruction, while full-blown intravascular hemolysis mediated by the terminal complement pathway is usually not prominent [4, 7, 31]. The explanation for this is probably the modest activation of the complement pathway, combined with the protective effect of the physiological cell surface complement inhibitors CD55 and CD59 which, unlike in PNH, are intact in AIHA.

**Figure 2:**
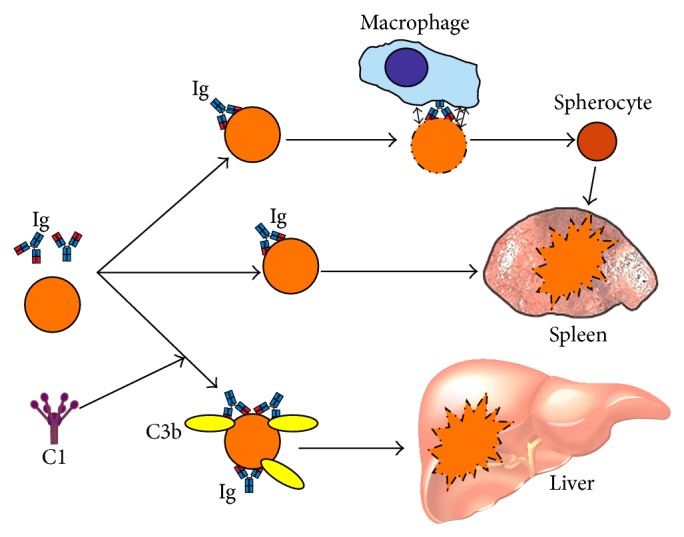
Mechanisms of erythrocyte destruction in warm-antibody autoimmune hemolytic anemia. Ig: immunoglobulin; C: complement protein.

The pathways of RBC destruction in w-AIHA are summarized in Figure 2. In conclusion, complement activation does occur to some extent, at least in a proportion of the patients, but is hardly essential for hemolysis in w-AIHA. DAT positivity for C3 fragments is a marker of complement involvement.

### 2.3. Therapy

The cornerstone of established pharmacological therapy for w-AIHA is unspecific immunosuppression. Corticosteroids remain first-line treatment. However, high initial doses are required, responses are often achieved slowly, and the rate of sustained remissions following weaning of steroids is only 15–30% unless second-line therapy is administered [3, 4, 36]. Combination with rituximab in the first-line setting has been shown to significantly increase response rate and duration [36], although rituximab has not gained general acceptance by most authors as part of first-line therapy [37]. The concern of adverse effects of rituximab in the AIHA setting will be discussed below in the context of therapy for CAD.

Since most RBC destruction occurs in the spleen, it is not surprising that splenectomy is a reasonably efficient second-line treatment, resulting in response in about two-thirds of the patients [4]. An alternative, safe, and efficient second-line option, if not used as part of combination therapy in the first line, is infusions of rituximab [38]. In the third-line situation, one may use immunosuppressive drugs such as danazol, azathioprine, cyclophosphamide, or cyclosporine [3, 4, 39], although response rates are poorly documented and most publications are case reports or small retrospective series. High-dose cyclophosphamide or alemtuzumab has been used with success in refractory cases [37]. In w-AIHA secondary to lymphoproliferative diseases, therapy for the underlying disorder is often essential and should be considered at an early stage. The future possibilities of complement-directed therapy will be addressed below. Transfusion in w-AIHA is an important and complex issue which has been comprehensively discussed elsewhere [4, 40, 41]. To avoid severe transfusion reactions and alloimmunization with increasing transfusion problems, indications should be restrictive and specific precautions undertaken. The risk of alloimmunization should be reduced by extended blood group phenotyping or the use of prophylactic antigen-matched donor blood and a bed-side biological compatibility test should be performed [40, 42, 43].

## 3. Primary Chronic Cold Agglutinin Disease

### 3.1. Etiology and Pathogenesis

We should distinguish between primary cold agglutinin disease (CAD) and secondary cold agglutinin syndrome (CAS) [5]. As will be further explained below, CAD is a precisely defined clinicopathological entity and should, therefore, be called a disease, not syndrome [44]. Secondary CAS, on the other hand, is a syndrome complicating a variety of infectious and neoplastic disorders, not a well-defined disease. In a Norwegian population-based study, the prevalence of CAD was 16 per million and the incidence was about 1 per million per year, making CAD account for approximately 15% of AIHA [1, 2, 45].

Cold agglutinins (CA) are autoantibodies that agglutinate RBCs with a temperature optimum of 3-4°C but may also act in a warmer environment, depending on the thermal amplitude of the CA [5, 46]. If the thermal amplitude exceeds 28–30°C, the CA will be pathogenic. Low-affinity CA also occur in many healthy individuals; these nonpathogenic CA are polyclonal, have low thermal amplitude, and are present in low titers, not higher than 256 and usually lower than 64. More than 90% of pathogenic CA are of the IgM class and these IgM macromolecules can be pentameric or hexameric [45, 47, 48].

In general, monoclonal CA are more pathogenic than polyclonal CA and hexameric IgM is more pathogenic than pentameric IgM [5, 48, 49]. It has been known for decades that, in patients with CAD, IgM-antibodies with CA-activity are monoclonal and, in more than 90% of the patients, show *κ* light chain restriction [50]. Accordingly, CAD patients must have a clonal B-cell lymphoproliferative disorder which has not been fully elucidated until the last years. Two large, retrospective studies of consecutive patients with primary CAD found signs of a bone marrow clonal lymphoproliferation in most patients, but in both series the individual hematological and histological diagnoses showed a striking heterogeneity [45, 51]. In one of the series, lymphoplasmacytic lymphoma (LPL) was the most frequent finding, while marginal zone lymphoma (MZL), unclassified clonal lymphoproliferation, and reactive lymphocytosis were also frequently reported [45]. The explanation for this perceived heterogeneity was probably revealed by a recent study in which bone marrow biopsy samples and aspirates from 54 patients with CAD were systematically reexamined by a group of lymphoma pathologists, using a standardized panel of morphological, immunohistochemical, flow cytometric, and molecular methods [44]. The bone marrow findings in these patients were consistent with a surprisingly homogeneous disorder termed “primary CA-associated lymphoproliferative disease” by the authors and distinct from LPL, MZL, and other previously recognized lymphoma entities. The MYD88 L265P somatic mutation, typical for LPL, could not be detected in the samples from patients with CAD [44, 52].

### 3.2. Mechanisms of Erythrocyte Destruction

**Figure 3:**
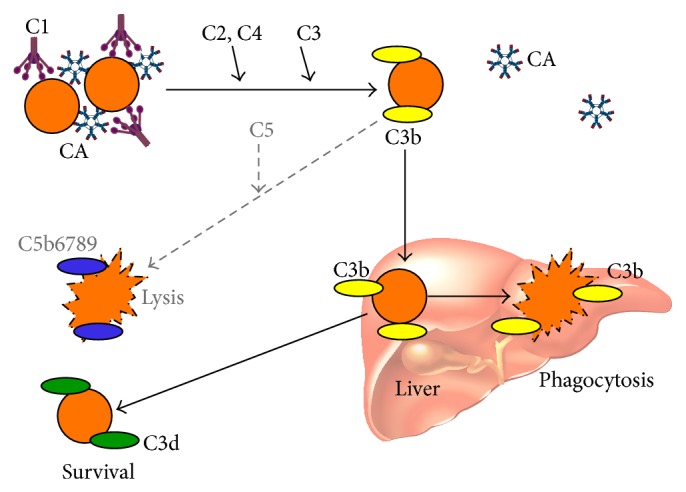
Immune-initiated, complement-mediated erythrocyte destruction in cold agglutinin disease (CAD) and cold agglutinin syndrome (CAS). See text for further explanation. Black arrows: major pathway; gray/dotted arrows: minor pathway; CA: cold agglutinin; C: complement protein.

CA are usually directed against the Ii blood group system, most CA in CAD being specific for the I carbohydrate antigen [53–55]. Cooling of blood during passage through acral parts of the circulation allows CA to bind to RBC and cause agglutination (Figure 3). Being a strong complement activator, antigen-bound IgM-CA on the cell surface binds C1 and thereby initiates the classical complement pathway [8, 56, 57]. C1 esterase activates C4 and C2, generating C3 convertase, which results in the cleavage of C3 to C3a and C3b. Upon returning to central parts of the body with a temperature of 37°C, IgM-CA detaches from the cell surface, allowing agglutinated erythrocytes to separate from each other, while C3b remains bound. A proportion of the C3b-coated RBCs is sequestered by macrophages of the reticuloendothelial system, mainly Kupfer cells in the liver. On the surface of the surviving RBCs, C3b is cleaved, leaving high numbers of C3d molecules on the cell surface. These mechanisms explain why the monospecific DAT is strongly positive for C3d in patients with CA-mediated hemolysis and, in the majority, negative for IgM and IgG [45].

Complement activation may proceed beyond the C3b formation step, resulting in C5 activation, formation of the membrane attack complex (MAC), and intravascular hemolysis. Due to surface-bound regulatory proteins such as CD55 and CD59, however, the complement activation is usually not sufficient to produce clinically significant activation of the terminal complement pathway. The major mechanism of hemolysis in stable disease, therefore, is the extravascular destruction of C3b-coated erythrocytes [10, 31, 57]. Obviously, however, C5-mediated intravascular hemolysis does occur in severe acute exacerbations and in some profoundly hemolytic patients, as evidenced by the finding of hemoglobinuria in 15% of the patients and the observation of a beneficial effect of C5 inhibition in at least occasional patients [45, 51, 58, 59].

Febrile infections, major trauma, or major surgery can result in acute exacerbation of hemolytic anemia in at least two-thirds of patients with CAD [45, 58, 60]. The explanation for this paradoxical exacerbation is that, during steady-state chronic disease, most patients are complement-depleted with low levels of C3 and often undetectable levels of C4. During acute phase reactions, C3 and C4 are repleted and exacerbation of complement-induced hemolysis ensues [55, 58].

### 3.3. Therapy

In textbooks and review articles, it is often postulated that typical patients with CAD are just slightly anemic and do not need pharmacological therapy. This holds true for a minority only; the median hemoglobin level in affected individuals is 9.0 g/dL and the lower tertile is 8.0 g/dL [45]. Furthermore, at least 90% of the patients experience cold-induced circulatory symptoms caused by RBC agglutination, most often in the form of acrocyanosis and/or Reynaud-phenomena that can range from slight to disabling [45]. In many patients, therefore, CAD is not an indolent disease in terms of major clinical symptoms and quality of life. In Norway as well as the United States, drug therapy had been attempted in 70–80% of unselected patients studied in two relatively large retrospective series [45, 51]. In contrast to w-AIHA, corticosteroids and other unspecific immunosuppressive drugs are of little or no value in CAD [45, 61].

The relative success in therapy for CAD during the last 10–12 years has been achieved by targeting the pathogenic B-cell clone [62]. Rituximab monotherapy has been shown in prospective studies to induce remission in approximately half of the patients, although complete remissions are unusual and the median response duration is only about 1 year [63, 64]. In both studies, events of cytokine-related reactions to rituximab were few and readily treatable. The studies found no significant problems with infectious complications due to the induced B-cell lymphopenia and hypogammaglobulinemia [45, 63, 64]. Data from rituximab maintenance in follicular lymphoma indicate that, in adults, even prolonged or repeated administration is safe with regard to infections [65]. Very rare cases of progressive multifocal leukoencephalopathy and hepatitis B reactivation have been reported, however, in patients receiving rituximab for polyclonal autoimmune disorders. Any causal associations are uncertain because of concomitant immunosuppressive therapies and immune dysregulation as part of the autoimmune disease itself [66].

In a more recent prospective trial, combined therapy with rituximab and fludarabine produced very high response rates (remission in 75% of the patients, including 20% complete remissions) and the median response duration was more than 66 months [67]. This regimen was, however, found to be significantly more toxic than rituximab monotherapy. No other immunochemotherapy regimens have been studied in published clinical trials. According to single case reports, favorable outcome has been observed following bortezomib-based regimens [68] and rituximab-bendamustine combination therapy [69].

Transfusion can safely be given in CAD provided specific precautions are undertaken, although these precautions are entirely different from those required in w-AIHA. Such requirements have been extensively described elsewhere [5, 70]. The perspective for future therapeutic use of complement inhibitors will be addressed below.

## 4. Secondary Cold Agglutinin Syndrome

Secondary CAS is far more uncommon than primary CAD. Among 295 consecutive individuals with AIHA described retrospectively by Dacie in a single-center series, 7 patients (2.4%) were classified as having CAS secondary to malignant disease [1]. CAS has been described in patients diagnosed with diffuse large B-cell lymphoma, Hodgkin's lymphoma, carcinomas, sarcomas, metastatic melanoma, and chronic myeloproliferative disorders [2]. Some of these associations have been poorly documented [5], and the most convincing association with malignant disease has been described with non-Hodgkin's lymphoma [71–74]. In CAS complicating aggressive lymphoma, the CA are monoclonal, most often IgM, and have anti-I specificity. In contrast to CA found in primary CAD, however, the light chain restriction can be *λ* as well as *κ* [71, 74].

Polyclonal anti-I specific CA of the IgM class are produced as part of the physiological immune response in* Mycoplasma pneumoniae* pneumonia. They do not usually give rise to significant hemolysis. In occasional patients, however, production of high-titer, high-thermal amplitude CA results in hemolytic anemia which is transient but can be severe [5, 75, 76]. CAS complicating* Mycoplasma pneumoniae* infection has been reported to account for approximately 8% of AIHA [1]. Still more uncommon but less severe, polyclonal anti-i specific CA of the IgM or IgG class can result in CAS in Epstein-Barr virus infection [5, 77]. Transient CAS has also been described following cytomegalovirus infection, varicella, rubella, adenovirus infection, influenza A,* Legionella pneumophila* pneumonia, listeriosis, and pneumonia caused by* Chlamydia* species [5].

In CAS secondary to infection or aggressive lymphoma, the RBC breakdown is complement-dependent, mediated by exactly the same mechanisms as in primary CAD (Figure 3) [5, 7]. Treatment of the underlying disease, if relevant and available, is often the only possible drug therapy for the hemolytic complication. Corticosteroid therapy has been used but is not evidence-based. In severely anemic patients, transfusions can safely be given provided the same precautions are carefully observed as in primary CAD [5].

## 5. Paroxysmal Cold Hemoglobinuria

In paroxysmal cold hemoglobinuria (PCH), polyclonal cold-reactive IgG-antibodies bind to the RBC surface protein antigen termed P but do not agglutinate the erythrocytes. The resulting hemolysis is entirely complement-dependent and the temperature optimum for complement activation is at 37°C [78, 79]. Such biphasic antibodies are called Donath-Landsteiner hemolysins. In Donath-Landsteiner's test, one sample of patient blood is incubated at 4°C and then at 37°C, while another sample is incubated at 37°C without having been preincubated in the cold [78, 79]. If biphasic autoantibodies are present, hemolysis will be observed only in the sample preincubated at 4°C. The sensitivity is limited because the patient blood is often complement-depleted, and, in more sensitive modifications of the test, complement is added and/or papain pretreated RBCs are used [79].

Fifty to 100 years ago, PCH was associated with tertiary syphilis, but this form is hardly seen anymore. In the 21th century, PCH occurs almost exclusively in children and accounts for 1–5% of childhood AIHA, making it a rare disease [80]. It appears as an acute, postinfectious complication, in most cases following a virus infection [79]. Single cases have also been reported in* Haemophilus influenzae* infection and, recently, visceral leishmaniasis [80, 81].

**Figure 4:**
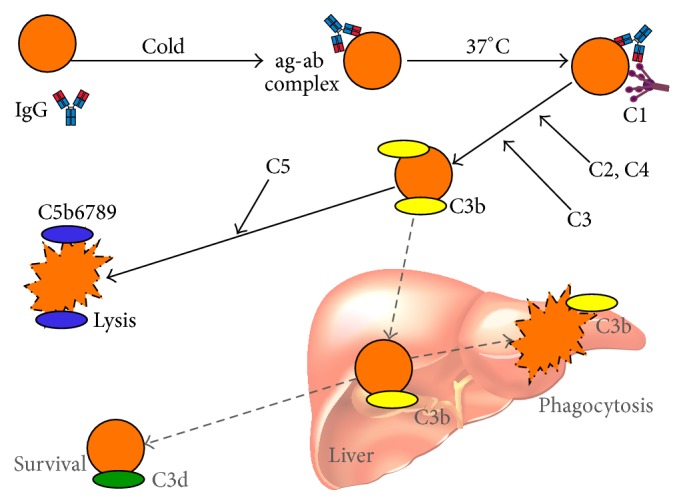
Immune-initiated, complement-mediated erythrocyte destruction in paroxysmal cold hemoglobinuria (PCH) showing a biphasic temperature optimum. Black arrows: major pathway (intravascular hemolysis); gray/dotted arrows: possible minor pathway; Ig: immunoglobulin; ag: antigen; ab: antibody; C: complement protein.

The P-anti-P complex is a very strong complement trigger, resulting in full-blown activation of the classical and terminal pathways (Figure 4). The hemolysis, therefore, is intravascular and massive; the onset is usually sudden and the clinical features include fever, pallor, jaundice, severe anemia, and macroscopic hemoglobinuria [79, 81]. Even though PCH is a transient complication with good prognosis, most patients will need transfusions, which can safely be given provided the same precautions are undertaken as in other cold-antibody AIHA [5]. Apart from therapy for any treatable underlying infection, no disease-modifying intervention has been documented. Although the administration of corticosteroids has been followed by improvement in some reported cases, the effect remains anecdotal and unproven [79, 81].

## 6. Mixed-Type Autoimmune Hemolytic Anemia

Mixed warm- and cold-antibody AIHA is probably very rare. The diagnostic work-up is complex and the condition is supposed to be overdiagnosed [82]. There are two obvious sources of error. First, patients with w-AIHA can, like healthy individuals, produce low-titer, low-thermal amplitude CA of no clinical significance. Second, up to 20% of patients with CAD have IgG on the RBC surface in addition to C3d [45, 82].

## 7. Complement Modulation for the Treatment of AIHA

### 7.1. Available Substances and Experimental Studies

The potential of pharmacological complement modulation for the treatment of AIHA will depend on (a) the type of AIHA and extent and level of complement involvement, (b) the availability, safety, and efficacy of complement-modulating drugs, and (c) the specific level of complement inhibition by these drugs. The search for targeted, therapeutic inhibitors of the complement cascade has been going on for 30–40 years, with few examples of success so far [9]. New* in vitro* and* in vivo* models for testing the impact of specific complement inhibition on immune hemolysis are, however, still being developed [10, 83].

Plasma-derived or recombinant C1-esterase inhibitor (C1-INH) has been available for decades and has been successfully used for the treatment of hereditary angioedema (HAE) [84]. Although not a complement-mediated disorder, HAE is caused by lack or deficiency of endogenous C1-INH and replacement therapy has been well studied. In AIHA, on the other hand, endogenous C1-INH production is normal, indicating that physiological concentrations of the inhibitor will not block complement-mediated hemolysis.

Eculizumab, a humanized monoclonal C5-antibody, has been shown to efficiently inhibit complement at the C5 level and, thereby, block the terminal pathway and prevent intravascular hemolysis by MAC. Therapy with eculizumab has been a great success in PNH, although complement-mediated hemolysis is not completely prevented [85]. The explanation for this is probably that patients with PNH lack physiological inhibitors both at a downstream level in the terminal pathway (CD59) and at an upstream level in the classical pathway (CD55). In consequence, a slight to moderate hemolysis mediated by phagocytosis of C3b-opsonized RBC will still occur along the same pathway as described in CAD, independent of C5 activation or inhibition [12].

Some newer complement-modulating drugs have been studied with promising results in preclinical experiments but not yet in the* in vivo* setting. Compstatin Cp40 is a low-molecular weight peptide complement inhibitor that blocks cleavage of C3 and has been found to efficiently prevent hemolysis of RBC from PNH patients* in vitro* [86].

**Figure 5:**
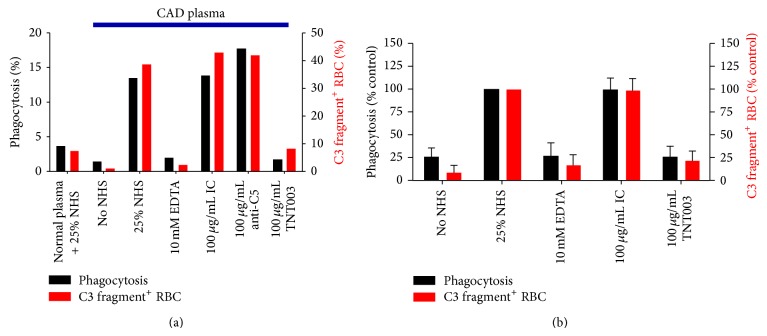
*In vitro* effects of anti-C1s antibody TNT003 on phagocytosis (black columns, left *y*-axis) and complement fragment deposition (red columns, right *y*-axis) on red blood cells after addition of normal human plasma and cold agglutinin-rich plasma (under blue bar), respectively. TNT003 inhibits phagocytosis and complement deposition, while anti-C5 has no impact. CAD: primary chronic cold agglutinin disease; NHS: normal human serum; IC: isotype control. Reproduced from Blood (Shi et al. 2014, [10]) with permission. Copyright: Blood, the Journal of the American Society of Hematology.

TNT003, a mouse monoclonal anti-C1s antibody (Figure 5), has recently been shown to completely inhibit* in vitro* hemolysis induced by CA [10]. This antibody targets C1s serine protease activity. Using CA samples from 40 patients with CAD, the authors found that TNT003 prevented CA-induced deposition of C3 fragments on the RBC at the same concentration of antibody that stopped hemolysis. Furthermore, C1s inhibition by TNT003 resulted in prevention of* in vitro* erythrophagocytosis by a phagocytic cell line. The classical-pathway-driven production of anaphylotoxins C4a, C3a, and C5a was also inhibited [10].

### 7.2. Complement Inhibition in Subtypes of AIHA: Future Perspective

In w-AIHA, as described above, complement activation plays some role but is not essential for pathogenesis in most patients. Complement modulation may be expected, therefore, to be of limited therapeutic value in w-AIHA in general and of no value if DAT is negative for C3 fragments. According to a recent, well-described case report, however, Wouters and colleagues observed favorable effect of plasma-derived C1-INH in a patient with a C3d-positive, therapy-resistant severe w-AIHA secondary to an aggressive non-Hodgkin's lymphoma [87]. Although very high doses of C1-INH were required, hemolysis was efficiently controlled and the efficacy of RBC transfusion dramatically improved following treatment. No other clinical observations on the results of complement inhibition have been published in w-AIHA. In patients with a positive DAT for C3d and very severe hemolysis, further studies of complement inhibition even at a more downstream level would be of interest, mainly as an attempt to temporarily control hemolysis.

Given that hemolysis in CAD is entirely complement-dependent, studies of complement inhibition would be relevant in CAD. A case report by Röth and coauthors described favorable effect of therapy with eculizumab [59]. This observation may seem somewhat surprising, since the predominant hemolytic pathway in CAD is not C5/MAC-mediated. A probable explanation is that activation of the terminal complement pathway does occur, after all, in acute exacerbations, in the chronic state of some severely affected patients and, possibly, as a minor pathway also in less severely affected patients. Further studies will be of interest.

In theory, complement inhibition at the C1 level should be very promising in CAD because this will block the classical-pathway-dependent, C3b-mediated extravascular hemolysis without compromising the alternative and lectin complement pathways. The published* in vitro* study of TNT003 is highly interesting, therefore, and it is to be hoped that a corresponding humanized antibody can be developed and further tested in the preclinical and clinical setting [10, 11].

Given that immunochemotherapy directed at the pathogenic B-cell clone is efficient and requires administration only for a limited period of time, do we actually need complement-modulating therapies for CAD? First, in at least 25% of the patients, immunochemotherapy is unsuccessful because of treatment failure or toxicity [67]. Second, rapidly acting therapies should be developed for some specific clinical situations, for example, acute severe exacerbations induced by infections, trauma, or major surgery and, possibly, before cardiac surgery in selected patients.

In the uncommon cases of CAS secondary to specific infection, there is often no need for therapy for the CAS* per se*. However, this is not always the case. Particularly in CAS following* Mycoplasma pneumoniae* pneumonia, the patients can be profoundly anemic and transfusion dependent for weeks until spontaneous resolution occurs [5]. Clinicians and patients would welcome a possibility for temporary control of this situation by complement inhibition along the same lines that may be developed in primary CAD. Systematic studies would be interesting but probably difficult to perform because of the rarity of the disorder.

In the rare cases of postinfectious PCH in children, measures for temporary control of the hemolysis will be valuable if such therapies can be developed. Since the terminal complement pathway is heavily involved, we do not necessarily need new substances; exploring the efficacy of eculizumab would be of great interest. Probably, however, prospective trials will never be performed because there are too few patients for such studies.

**Figure 6:**
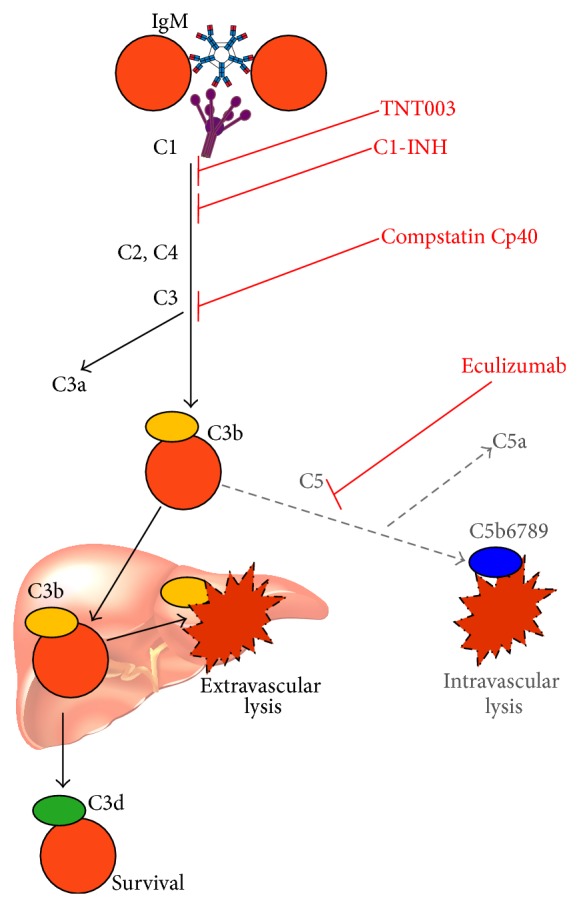
Future perspective on complement modulation in AIHA. Inhibitors and levels of inhibition. Black arrows: major pathway in cold agglutinin disease (CAD); gray/dotted arrows: minor pathway in CAD and major pathway in paroxysmal cold hemoglobinuria; Ig: immunoglobulin; C: complement protein; TNT003: mouse monoclonal C1s antibody; C1-INH: C1 esterase inhibitor. Previously published in Blood (Berentsen 2014, [11]), reused with permission. Copyright: Blood, the Journal of the American Society of Hematology.

Present and future possibilities for therapeutic complement inhibition in AIHA are summarized in Figure 6. It is important to ask whether such therapy will be dangerous. The complement system is, after all, an essential part of the innate immune system. Based on studies of eculizumab in PNH, we already have extensive information on the risk of severe infection following C5 inhibition. Provided the patients can be efficiently protected against meningococci, studies and clinical experience have shown that the risk of infection is negligible [85]. Complement inhibition at the C3 level may carry a much higher risk because efficient inhibition of C3 will completely block complement activation beyond this level, whether initiated by the classical, alternative, or lectin pathway [11, 86]. Interestingly, however, the still more proximal blockade at the C1 level achieved by TNT003 will selectively affect the classical pathway as required for control of hemolysis in CAD, while the lectin and alternative pathways will remain intact. Probably, therefore, these pathways will still enable the system to generate anaphylotoxins C3a and C5a in response to microbial stimuli, even though the production of these anaphylotoxins induced by the classical pathway will be blocked [10, 11]. Although this selectivity may, theoretically, reduce the risk of infection, careful studies will be required to address this issue.

## 8. Conclusion

The mechanism of RBC destruction differs considerably among the various types of AIHA, and so do the extent, level, and details of complement involvement. The theoretical background for therapeutic complement inhibition in selected subgroups of patients is very strong in CAD, CAS, and PCH but more limited in w-AIHA. Complement modulation is not an established or evidence-based therapy modality in any type of AIHA, but a number of experimental and preclinical studies are in progress and a few clinical observations have been reported. It will be important to carefully address safety issues. Clinical studies of new complement inhibitors are probably not far ahead.

## Figures and Tables

**Table 1:** Autoimmune hemolytic anemia.

Warm-antibody type	
Primary	
Secondary	
Cold-antibody type	
Primary chronic cold agglutinin disease	
Secondary cold agglutinin syndrome	
Associated with malignant disease	
Acute, infection-associated	
Paroxysmal cold hemoglobinuria	
Mixed cold- and warm-antibody type	
